# Microglia Contributes to BAF-312 Effects on Blood–Brain Barrier Stability

**DOI:** 10.3390/biom12091174

**Published:** 2022-08-25

**Authors:** Simona Federica Spampinato, Giuseppe Costantino, Sara Merlo, Pier Luigi Canonico, Maria Angela Sortino

**Affiliations:** 1 Department of Scienza e Tecnologia del Farmaco, University of Turin, Via P. Giuria 13, 10125 Turin, Italy; 2 Ph.D. Program in Neuroscience and Education, DISTUM, University of Foggia, 71121 Foggia, Italy; 3 Department of Biomedical and Biotechnological Sciences, Section of Pharmacology, University of Catania, Via Santa Sofia 97, 95123 Catania, Italy; 4 Laboratory of Neuroplasticity, Department of Pharmaceutical Sciences, University of Piemonte Orientale, 28100 Novara, Italy

**Keywords:** endothelial cells, siponimod, S1P1, HMC3 cells, CCL5, CCR5, claudin-5, barrier permeability

## Abstract

Microglia, together with astrocytes and pericytes, cooperate to ensure blood–brain barrier (BBB) stability, modulating endothelial responses to inflammatory insults. Agonists of the sphingosine 1 phosphate (S1P) receptors, such as siponimod (BAF-312), are important pharmacological tools in multiple sclerosis and other inflammatory diseases. Modulation of S1P receptors may result in a reduced inflammatory response and increased BBB stability. An in vitro BBB model was reproduced using human-derived endothelial cells, astrocytes and microglia. Co-cultures were exposed to inflammatory cytokines (TNFα, 10 UI and IFNγ, 5 UI) in the presence of BAF-312 (100 nM), and the BBB properties and microglia role were evaluated. The drug facilitated microglial migration towards endothelial/astrocyte co-cultures, involving the activity of the metalloprotease 2 (MMP2). Microglia actively cooperated with astrocytes in the maintenance of endothelial barrier stability: in the triple co-culture, selective treatment of microglial cells with BAF-312 significantly prevented cytokines’ effects on the endothelial barrier. In conclusion, BAF-312, modulating S1P receptors in microglia, may contribute to the reinforcement of the endothelial barrier at the BBB, suggesting an additional effect of the drug in the treatment of multiple sclerosis.

## 1. Introduction

The blood–brain barrier (BBB) is a fundamental structure in our body that maintains and protects the brain from external insults. The proper functions of the BBB and its ability to respond to environmental changes in physiological and pathological conditions are strictly modulated by the crosstalk between different structures. Endothelial cells, pericytes and astrocytes are the main constituents of the BBB, and they interact with the components of the neurovascular unit (NVU) that involves neuronal cells, microglia and the extracellular matrix [1]. NVU components directly communicate with endothelial cells, which can dynamically respond to pathological stressors [2]. Microglia cells represent the central nervous system (CNS)’s resident immune cells. In physiological conditions, microglia exhibit small cellular movements and extend radial processes from the cell body to patrol the surrounding environment [3]. Pathological mediators or traumatic events may quickly activate microglia in response to CNS insult. The nature of microglial responses may vary: they can remove cellular debris in an attempt to contribute to cellular repair, or may precipitate the induction of the neuroinflammatory response [4,5,6,7]. As part of the NVU, microglial cells may modify BBB properties [8]. Resting microglia have been described in close proximity to cerebral vessels, allowing constant microglial evaluation of BBB integrity, as well as of the influx of potentially noxious agents towards the CNS [9]. As we have already described for astrocytes [10], microglia also contribute to the maintenance of BBB properties in physiological conditions [11], and they can critically support the formation of barrier tight junctions in inflammatory conditions [12]. Accordingly, microglia depletion can facilitate BBB permeability [13]. On the contrary, sustained inflammation can trigger microglia to induce BBB damage [12]. Increased cyclooxygenase 2 activity in reactive microglia can facilitate the release of inflammatory cytokines (TNFα, IL1β, IL6) and the activation of pathways that contribute to BBB destabilization [14,15,16]. Thus, as observed in neuronal damage and also in the interaction with the cellular constituents of the BBB, microglia may play a dual role, initially protecting BBB integrity, but enabling its enhanced permeability during prolonged inflammation [12].

The BBB may be affected in several neurological disorders, and its alterations are pathognomonic in multiple sclerosis (MS), a neurodegenerative disorder where a leaky BBB is often visualized in MR imaging and in postmortem brains [17]. One of the possible interventions in the treatment of MS is the modulation of sphingosine 1 phosphate (S1P) receptors. S1P is a bioactive sphingolipid that modulates several physiological and pathological processes by acting on five receptors (S1P1-S1P5), broadly expressed in several tissues and districts of the body [18]. Acting as a functional S1P1 antagonist, fingolimod, the first-in-class S1P1/S1P5 modulator, prevents leukocytes’ egress from the lymph nodes [19]. Nevertheless, the anti-inflammatory role played by S1P modulators has been widely described [20]. Our group and others have demonstrated that fingolimod and siponimod (BAF-312), the recently approved S1P1/5 modulators for the treatment of secondary progressive MS, are able to stabilize the BBB, acting on both endothelial cells and astrocytes [21,22,23].

Supported by preclinical evidence indicating consistent brain penetration/distribution for siponimod across species [24], we decided to evaluate the effects that BAF-312 exerts on the CNS-resident immune cells, microglia, and whether these could affect the BBB properties.

## 2. Materials and Methods

### 2.1. Reagents and Materials

Tumor necrosis factor-α (TNFα) and interferon-γ (IFNγ) were purchased from Peprotech (London, UK), and JTE-013 from Sigma-Aldrich (St Louis, MO, USA). GM-6001, MMP-2 inhibitor III and MMP-9 inhibitor I were obtained from Calbiochem, Merck Millipore (Darmstadt, Germany). BAF-312 (BAF), NIBR-0213 (NIBR) and UC-42-WP04 (UC) were provided by Novartis Pharma (Basel, Switzerland). Collagen-I rat tail was provided by Corning (Milan, Italy). All cell culture plastics were from BD Falcon (Milan, Italy). Polycarbonate transwell inserts (0.4, 3 and 8 μm) were purchased from Corning, Merck Millipore.

### 2.2. Cell Cultures

For all experiments, cell lines at passage between 18 and 24 were used. The HMC3 human microglial cell line was purchased from the American Type Culture Collection (ATCC, LGC Standards, Manassas, VA, USA). Cells were grown in Eagle’s Minimum Essential Medium (EMEM; Merck Millipore) supplemented with 10% fetal bovine serum (FBS; Thermo Fisher Scientific, Milan, Italy) adding sodium pyruvate (1X; Thermo Fisher Scientific), non-essential amino acids (1X; Thermo Fisher Scientific) and penicillin (100 /mL)/streptomycin (100 μg/mL; Thermo Fisher Scientific) at 37 °C and in a 5% CO_2_ atmosphere. Cell lines used to simulate the BBB were adult human immortalized cells. Both the brain microvascular endothelial TY-10 cells and hAST astrocytic cells were developed at Yamaguchi University (Yamaguchi, Japan) in the laboratories of Dr. Sano and Kanda; both were transfected with a plasmid expressing temperature sensitive Simian virus-40 large T-antigen and the catalytic subunit of human telomerase, as previously described [25]. TY-10 cells were grown in MCDB-131 media (Thermo Fisher Scientific), supplemented with EGM-2 SingleQuots (Lonza, Basel, Switzerland) and 20% heat-inactivated fetal bovine serum (FBS Thermo Fisher Scientific). hAST were grown in astrocyte medium containing 2% heat-inactivated FBS, astrocyte growth supplement and penicillin/streptomycin solution, as provided with the Astrocyte Media Kit (Clinisciences, Nanterre, France). Both cell lines grew at 33 °C for two days and then endothelial cells and astrocyte cultures were placed together and transferred to 37 °C, where they exhibited growth arrest and differentiation. After two days at 37 °C, microglial cultures were added to the double endothelial/astrocyte co-cultures and exposed to treatments. All co-cultures and treatments were performed in astrocyte medium. All experiments were carried out in the presence of S1P2 antagonist JTE-013 (1 µM). Treatment with cytokines (TNFα, 10 IU and IFNγ, 5 IU; TI) was maintained for 24 h. When required by the experimental design, cultures were pretreated with NIBR (1 µM), GM-6001 (5 µM), MMP-2 inhibitor III (200 nM) and MMP-9 inhibitor I (200 nM) for 30 min and BAF (100 nM) or UC (1 µM) for 15 min prior to TI exposure and were then maintained in association with the cytokines for a further 24 h. To obtain endothelial/astrocyte-conditioned medium (BBB_CM), astrocyte/endothelial co-cultures were exposed to either vehicle (BBB_CM_ctr), TI (10 UI and 5 UI; BBB_CM_ TI) or TI + BAF (100 nM, BBB_CM_BAF + TI) for 6 h. Cultures were then washed and incubated with fresh medium for a further 18 h. This protocol was chosen in order to allow the exposure of astrocyte/endothelial co-cultures to TI and BAF for a time period sufficient to induce a BBB response before removal, and it has been already described [21,26]. CM was then collected and transferred to HMC3 cultures that were exposed to TI or BAF + TI for a further 24 h.

### 2.3. FITC–Dextran Permeability Assay

HMC3 cells (100 k/well) were plated on 24-well plates and grown at 37 °C for 2 days before treatment. hAST (90 k/each) were seeded on coverslips (Φ 12 mm; Menzel-Glaser, Thermo Fisher Scientific) supported by paraffin wax spacers into 24-well plates. After 1 h, polycarbonate transwell tissue culture inserts were transferred to the same plates and TY-10 cells were plated on top of the insert (0.33 cm^2^ inserts, 120 k/each) and cultures were grown in astrocyte medium. After two days at 33 °C, cells were transferred at 37 °C to allow cell differentiation for two more days. On day 3, endothelial/astrocyte co-cultures were added to HMC3 cells and co-cultures were exposed to treatment in AC medium for 24 h. When indicated, HMC3 cells were pre-exposed to TI (10 UI, 5 UI, respectively) or BAF + TI (BAF 100 nM, TI 10 UI and 5 UI, respectively). After 24 h, pretreatment was washed and endothelial/astrocyte co-cultures were added to HMC3 cells. Triple co-cultures were then exposed either to TI or BAF + TI according to the experimental protocol. After 24 h, inserts, containing the endothelial monolayer, were equilibrated in the “permeability medium” (phenol red-free Dulbecco’s modified Eagle’s medium, Thermo Fisher Scientific, supplemented with 1% FBS) for 30 min at 37 °C. Solute permeability was assessed using 10 kDa fluorescein isothiocyanate (FITC)-conjugated dextran (5 mg/mL, Sigma-Aldrich). Dextran was added to the luminal compartment. From the abluminal compartment at different time points (30 and 60 min), 100 µL of sample was collected and fluorescence was measured at 485/520 nm (excitation/emission) using a Varioskan TM LUX multimode microplate reader (Thermo Fisher Scientific). Fluorescence intensity values were plotted on the Y axis and represented as % of control.

### 2.4. Scratch Wound Assay

HMC3 cells (150 k/well) were plated on 24-well plates and grown at 37 °C for 2 days. When confluence was reached, the monolayer was scratched with a sterile P200 pipette tip according to a paradigm previously described [27,28]. After removal of the resulting debris by repeated washes, cells were subjected to treatment in AC medium, and scratch wound closure was monitored by phase microscopy, capturing images of the same field with a 10X objective at different times (8 h and 24 h). When specified, HMC3 cells were exposed to treatment in the presence of medium derived from astrocytic/endothelial co-cultures (BBB_CM), as previously described. The cell-free area was determined with the aid of the image processing software “Image J” developed by NIH and in public domain.

### 2.5. Transwell Migration Assay

Microglial migration towards the BBB was evaluated using a six-well transwell chamber (Corning). Astrocytes (300 k) were plated on coverslips (22 mm × 22 mm; Menzel-Glaser) supported by paraffin wax spacers, while endothelial cells (500 k/well) were plated on six-well plates. Endothelial/astrocyte co-cultures, simulating the BBB, were grown at 33 °C for two days and were transferred at 37 °C to allow cell differentiation for two more days. On the third day, transwell chambers containing microglia were added. HMC3 cells (1000 k/insert) were plated in the upper chamber of an 8 μm pore size insert in the six-well plate and allowed to migrate towards endothelial/astrocyte co-cultures, present in the lower chamber. Both endothelial/astrocyte co-cultures and HMC3 cells were exposed to treatments as indicated. Cells were incubated for 24 h, and then the non-migrating cells of the upper chamber were removed with the aid of a cotton swab, and the cells that had migrated to the lower surface of the membrane were stained with hematoxylin. The number of migrated cells was determined in 6 representative fields and counted using ImageJ software.

### 2.6. Western Blot

Endothelial cells (500 k/well) were plated on six-well plates. Astrocytes (300 k) were plated on coverslips (22 mm × 22 mm; Menzel-Glaser) supported by paraffin wax spacers, and HMC3 cells (800 k/insert) were plated on 0.4 μm pore poly-carbonate membrane transwell inserts (Falcon). This setting allowed the passage of soluble factors between endothelial cells, astrocytes and microglia but not their direct physical contact, thus also facilitating their isolation for subsequent investigations. When indicated, HMC3 cells were pre-exposed to treatment for 24 h and then washed and added to endothelial/astrocyte co-cultures. Cultures were then exposed to treatments for 24 h. After this, endothelial and microglial pellets were collected and lysed in RIPA lysis buffer (Sigma-Aldrich) supplemented with protease and phosphatase inhibitors.

Forty μg of each sample were separated by sodium dodecyl sulfate PAGE and transferred to a nitrocellulose membrane. Membranes were blocked with Blocker™ FL Fluorescent Blocking Buffer (Thermo Fisher Scientific). The following primary antibodies were used: rabbit anti-Claudin-5 (1:300; Thermo Fisher Scientific), rabbit anti-MMP9 (1:700, Millipore Merck), mouse anti-GAPDH (1:1000; Millipore Merck). After incubation, membranes were processed for immunodetection using specific fluorescent AlexaFluor 488 Plus-conjugated secondary antibody (Thermo Fisher Scientific) and IRdye 800 secondary antibody (Licor). Fluorescent signals were detected using IBright 1500 (Thermo Fisher Scientific). Band intensity was analyzed using the image processing software “ImageJ” developed by NIH and in the public domain.

### 2.7. Gelatin Zymography Assay

HMC3 cells (800 k/well) were plated on six-well plates and, when confluence was reached, the monolayer was subjected to mechanical damage by multiple scratches using a sterile P200 pipette tip. After 24 h treatment, pellets were collected in RIPA lysis buffer (Sigma-Aldrich) supplemented with protease and phosphatase inhibitors. Fifty μg of each sample was separated in non-reducing conditions using Novex 10% Zymogram (Gelatin) Gels (Thermo Fisher Scientific), containing gelatin as a substrate. The gel was washed in renaturing buffer (2.5% TritonX-100) for 45 min at 25 °C and was then incubated with zymography development buffer (Bio-Rad Laboratories, Milan, Italy) for 45 min at 25 °C and maintained in fresh zymography development buffer for 22 h at 37 °C. The gel was then stained with 0.25% R-250 Coomassie solution (Sigma; 30 min at 25 °C) and destained in a 2:1 methanol:acetic acid solution (30 min at 25 °C). IBright 1500 (Thermo Fisher Scientific) was used to acquire digital scans of the gels. Images were subjected to densitometric analysis with the aid of the ImageJ processing software (https://imagej.nih.gov accessed on 2 August 2022).

### 2.8. Quantitative Real-Time Polymerase Chain Reaction (PCR)

Astrocytes (300 k) were plated on coverslips (22 mm × 22 mm; Menzel-Glaser), supported by paraffin wax spacers and co-cultured with endothelial cells (500 k/well), plated on six-well plates, and HMC3 cells (800 k/insert), plated on 0.4 μm pore poly-carbonate membrane transwell inserts (Falcon), allowing the passage of soluble factors and facilitating astrocyte/endothelial selective isolation. HMC3 cells (800 k/well) were plated on six-well plates and, when confluence was reached, the monolayer was subjected to mechanical damage by multiple scratches using a sterile P200 pipette tip. After 24 h treatment, total RNA was extracted from either astrocyte or microglia cell culture using the RNeasy Plus Micro Kit (Qiagen, Milan Italy). One μg of RNA was used for cDNA synthesis, using the Superscript-VILO kit (Thermo Fisher Scientific). Quantitative RT-PCR was performed with Rotor-Gene Q using the QuantiNova SYBR Green PCR Kit (Qiagen). The melting curves obtained after each PCR amplification reaction confirmed the specificity of the 2-[*N*-(3-dimethylaminopropyl)-*N*-propylamino]-4-[2,3-dihydro-3-methyl-(benzo-1,3-thiazol-2-yl)-methyli-dene]-1-phenyl-quinolinium (SYBR Green assays). The following pairs of primers were used for human CCL 5 (F 5′- CCATGAAGGTCTCCGCGGCAC-3′, R 5′- CCTAGCTCATCTCCAAAGAG-3′) and human MMP2 (F 5′-AGATCTTCTTCTTCAAGGACCGGTT-3′, R 5′-GGCTGGTCAGTGGCTTGGGGTA-3′) as described [26,29] (all from Invitrogen, Thermo Fisher Scientific). Human CCR5 (QT00998802) and human RPLP0 (QT00075012), used as an endogenous control, were primeQuantitec from Qiagen. Expression fold changes were calculated by applying the 2–ΔCt method.

### 2.9. Statistical Analysis

All data are expressed as mean ±SEM of 3–5 different experiments, each run in duplicate or in triplicate, as specified in the figure legends. Data were analyzed by one-way ANOVA, followed by the Newman–Keuls test for significance. *p* < 0.05 was taken as the criterion for statistical significance.

## 3. Results

### 3.1. BAF-312 Induces Microglia Migration

**Figure 1:**
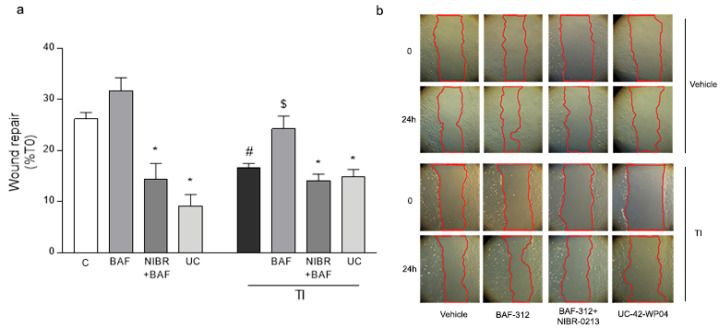
**Microglia migration is induced by BAF-312**: HMC3 were exposed to BAF-312 (BAF, 1oo nM), NIBR-0213 (NIBR, 1 μM), UC-42-WP04 (UC, 1 μM), either alone or in the presence of inflammatory cytokines, TNFα (10 IU) and IFNγ (5 IU; TI), for 24 h. (**a**) The percent closure of the empty area at 24 h vs. time 0 (T0; a) and representative images of the wound repair over time (**b**) are shown. Data are mean ± SEM of 4 independent experiments each run in triplicate. * p < 0.05 versus proper BAF, # *p* < 0.05 versus control (c), $ *p* < 0.05 vs. BAF. Significance was assessed by one-way ANOVA followed by Newman–Keuls test.

HMC3 cells were cultured in 24-well plates. When confluence was reached, the monolayer was subjected to a scratch wound migration assay, in which the ability of cells to migrate and cover the empty space was monitored. Migration was monitored for up to 30 h. Untreated HMC3 cells started migrating after 5 h (10% mean wound closure, data not shown), but we decided to analyze the nature of cell migration at 24 h, when, in untreated conditions, it reached almost 30% of closure (Figure 1a,b). TNFα + IFNγ (TI; 10 UI and 5 UI, respectively) significantly reduced HMC3 cells’ migration. The S1P1/5 agonist BAF did not affect HMC3 cells’ migration in resting conditions, but it significantly prevented TI effects. BAF activity on HMC3 cells’ migration appeared to be mediated by S1P1. Both in resting conditions and after exposure to TI, in fact, the S1P1 antagonist NIBR-0321 (NIBR, 1 μM) prevented HMC3 cells’ movement (Figure 1a,b). Similarly, the selective S1P5 agonist, UC-42-WP04 (UC, 1 μM), blocked basal HMC3 cell migration.

### 3.2. HMC3 Mobility Is Mediated by MMP2

**Figure 2:**
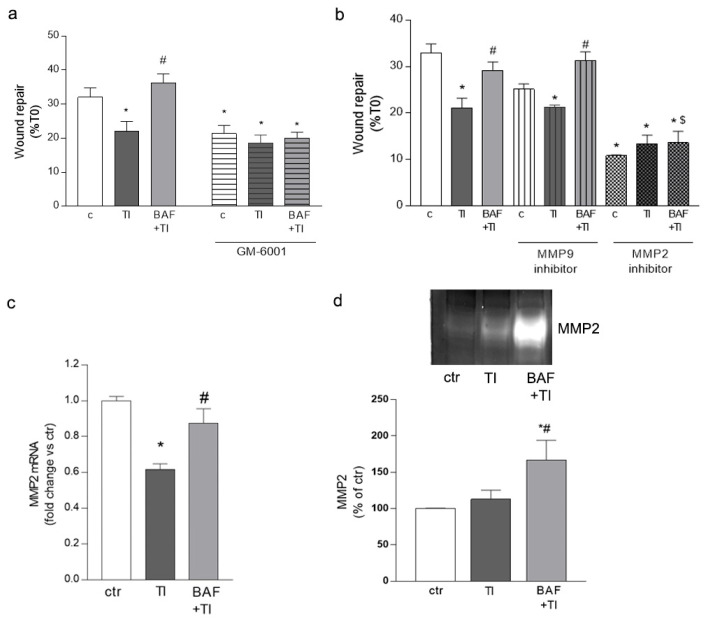
**BAF-312 drives HMC3 cell migration through MMP2**. The involvement of MMPs in microglial migration was assessed by evaluating the ability of HMC3 cells to migrate into the empty area and to repair the wound for 24 h in the presence of the non-selective MMP inhibitor (GM 6001, 5 μM) (**a**) or the selective MMP2 and MMP9 inhibitors (both at 200 nM) (**b**). HMC3 cells were subjected to multiple scratches in the presence of BAF-312 (BAF, 100 nM) and TI (10 UI and 5 UI, respectively); gene expression (**c**) and enzymatic activity (**d**) of MMP2 were evaluated after 24 h. In (**d**), representative blots and densitometric analysis are reported. Data are mean ± SEM of 3 independent experiments each run in triplicate (**a**,**b**) and 3 independent experiments (**c**,**d**). * *p* < 0.05 versus ctr, # *p* < 0.05 versus TI, $ *p* < 0.05 vs. BAF + TI. Significance was assessed by one-way ANOVA followed by Newman–Keuls test.

Microglial migration may be mediated by the release of extracellular matrix proteases such as metalloproteases (MMPs). Thus, we exposed HMC3 cells to inflammatory cytokines TI (10 UI and 5 UI), either alone or with BAF (100 nM), in the presence of the non-selective MMP inhibitor GM-6001 (GM, 5 μM, added 20 min before BAF exposure). Treatment with GM completely prevented HMC3 cell migration in all the conditions investigated, implying that microglial mobility strongly relies on MMP activity (Figure 2a). The specific involvement of the two gelatinases, MMP2 and MMP9, in HMC3 cell migration was investigated using selective inhibitors (MMP2 inhibitor III and MMP9 inhibitor I, both at 200 nM). The MMP9 inhibitor only slightly affected HMC3 cell migration in resting conditions and did not modify the ability of BAF to counteract the TI-induced reduction in migration (Figure 2b). On the contrary, the MMP2 inhibitor prevented HMC3 cell migration, either alone or in the presence of TI and BAF (Figure 2b), thus reinforcing the idea that microglial migration is strongly dependent on MMP2 activity, and BAF may modulate the activity of this protease. Gene expression of MMP2 and its activity were then evaluated on microglial cells by RT-PCR and the enzymatic zymography assay. Microglia cells were plated and, when confluence was reached, the monolayer was subjected to multiple scratches, to evaluate MMP2 expression and activity under conditions of stimulated migration. Cultures were then exposed to TI (10 UI and 5 UI), either alone or in the presence of BAF (100 nM). TI exposure reduced the mRNA levels of MMP2 and this effect was counteracted by BAF pretreatment (Figure 2c). Accordingly, in the presence of BAF, increased MMP2 enzymatic activity was observed as by gel zymography (Figure 2d).

### 3.3. HMC3 Cells’ Mobility towards Endothelial/Astrocyte Co-Cultures Is Facilitated by BAF-312

**Figure 3:**
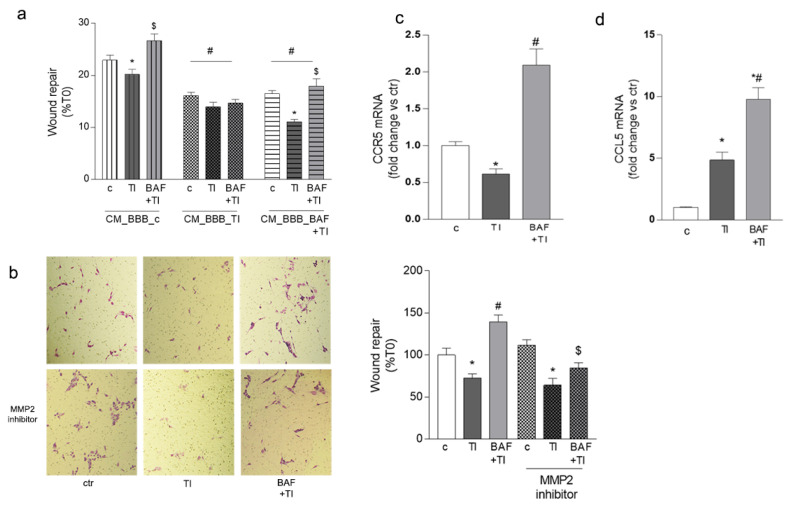
**BAF-312 facilitates microglia movement towards the BBB.** BAF-312′s (BAF, 100 nM) ability to influence microglia migration towards the BBB in the presence of TI (10 UI, 5 UI) was investigated by the scratch wound assay (**a**), where HMC3 cells were exposed to treatment in the presence of conditioned medium (CM) derived from endothelial/astrocyte co-cultures in resting condition (CM_BBB_c) or pre-exposed to TI (CM_BBB_TI) or BAF + TI (CM_BBB_BAF + TI). In (**b**), evaluation of the number of HMC3 cells migrating through an 8 μm pore transwell insert towards endothelial/astrocyte co-cultures after exposure to TI or BTI in the presence of the MMP2 inhibitor III (200 nM) is shown. A representative image of migrated cells and related plots are shown (**b**). HMC3 mRNA gene expression of CCR5 (**c**) and astrocytic mRNA gene expression of CCL5 (**d**) after 24 h exposure to TI and BAF + TI. Data are mean ± SEM of 3 independent experiments each run in triplicate (**a**) or in duplicate (**b**) and 3 independent experiments (**c**,**d**). * *p* < 0.05 vs. proper ctr, # *p* < 0.05 vs. proper TI, $ *p* < 0.05 vs. BAF + TI. Significance was assessed by one-way ANOVA followed by Newman–Keuls test.

We wondered whether HMC3 cells’ movement could be driven by their crosstalk with other cell types, and more specifically with the cellular components of the BBB. For this reason, we first evaluated whether exposure to medium derived from endothelial/astrocyte co-cultures could affect microglia migration. Endothelial/astrocyte co-cultures, simulating an in vitro BBB, were exposed to TI (10 UI and 5 UI) or BAF + TI (BAF, 100 nM and TI 10 UI and 5 UI, respectively) for 6 h; they were washed to remove treatments and fresh medium was added for a further 18 h to obtain conditioned medium (CM), which was collected. HMC3 cells were exposed to CM derived from resting BBB (CM_BBB_c), or from BBB previously exposed to TI (CM_BBB_TI) or BAF and TI (CM_BBB_BAF + TI). Under these conditions, HMC3 cells were treated with TI (10 UI and 5 UI) or BAF + TI (BAF 100 nM and TI 10 UI and 5 UI, respectively) and subjected to a scratch wound migration assay. The mobility of HMC3 cells was not influenced by the exposure to CM derived from resting BBB: in this condition, as expected, BAF counteracted the TI-induced reduction in microglial migration (Figure 3a). On the contrary, HMC3 cells’ exposure to both CM_BBB_TI and CM_BBB_BAF + TI reduced HMC3 cells’ migration. BAF, directly added to HMC3 cells, was able to prevent the reduced migration induced by TI only in the presence of CM_BBB_BAF + TI, and not in microglia exposed to CM_BBB_TI (Figure 3a).

To further investigate proper microglial migration towards the BBB, we plated HMC3 cells on 8 μm pore transwell inserts and evaluated their movement towards the BBB culture. Both endothelial/astrocyte co-cultures and HMC3 cells were exposed to TI (10 UI and 5 UI) or BAF + TI (BAF 100 nM and TI 10 UI and 5 UI, respectively), and after 24 h, the number of migrated microglia, detected on the lower surface of the membrane inserts, was higher after staining. As observed in the scratch wound migration assay, TI reduced HMC3 cells’ migration towards the BBB, an effect significantly prevented by BAF exposure (Figure 3b, upper panel and graph). The MMP2 inhibitor (200 nM) was added to co-cultures 20 min before BAF. Inhibition of MMP2 activity, per se, did not affect the ability of HMC3 cells to migrate towards the BBB, while it significantly reduced BAF-induced movement, in the presence of cytokines, pointing out the major role of the protease in inflammatory conditions (Figure 3b, lower panel and graph).

Microglia migration towards the perivascular space may be driven in a CCR5-dependent manner [12]. For this reason, we evaluated the mRNA expression of CCR5 on microglia cells. While TI significantly reduced CCR5 expression, BAF pretreatment completely reverted TI’s effects (Figure 3c). Accordingly, we evaluated whether the expression of the CCR5 ligand, CCL5, was affected by BAF exposure. We decided to evaluate CCL5 mRNA expression in astrocytes, cells that, in the CNS, are known to strongly interact with microglia. Astrocytes co-cultured with endothelial cells and microglia were exposed for 24 h to TI either alone or in the presence of BAF. The exposure to the inflammatory stimulus, per se, was able to induce the astrocytic expression of CCL5, an effect significantly potentiated by BAF (Figure 3d).

### 3.4. HMC3 Cells Exposed to BAF-312 Modulate BBB Properties

**Figure 4:**
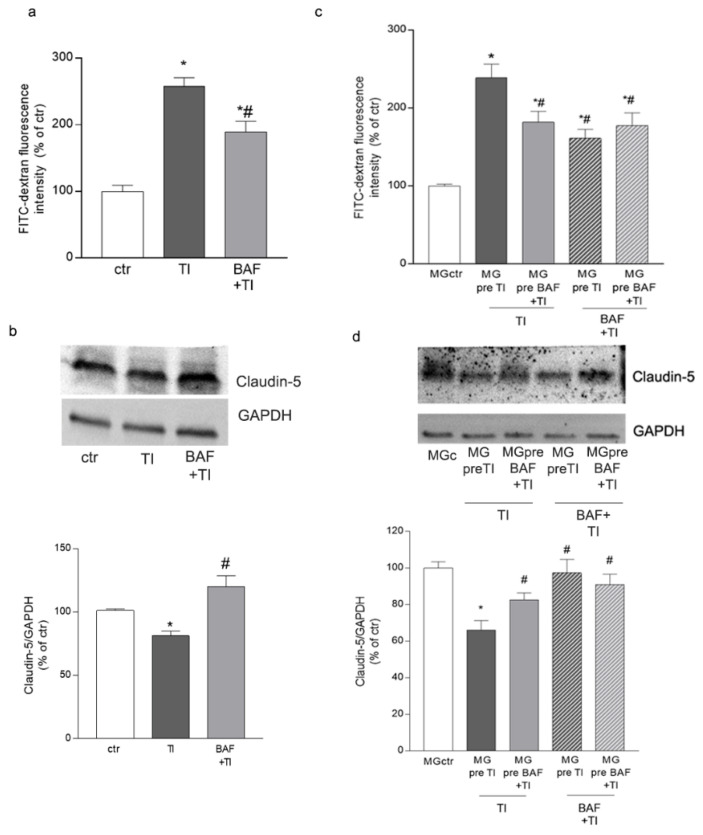
**BBB properties are modulated in the presence of BAF-312-treated microglia.** Endothelial permeability to FITC-conjugated dextran (**a**) and its expression of the junctional protein claudin-5 (**b**) were evaluated in a triple co-culture where endothelial cells, astrocytes and microglia were simultaneously exposed to TI (10 UI, 5 UI) or BAF + TI (100 nM, 10 UI and 5 UI, respectively) for 24 h. HMC3 were pre-exposed to TI (MG pre TI, 10 UI, 5 UI) or BAF + TI (MG pre BAF+ TI, 100 nM 10 UI and 5 UI, respectively) for 24, and then added to endothelial/astrocyte co-cultures. Triple co-cultures were then exposed to either TI (10 UI, 5 UI) or BAF + TI (100 nM, 10 UI and 5 UI, respectively), and endothelial permeability to FITC-conjugated dextran (**c**) and its expression of the junctional protein claudin-5 (**d**) were evaluated. Representative blots and densitometric analysis are reported (**b**,**d**). Data are mean ± SEM of 3 independent experiments each run in duplicate (**a**,**c**) and 3 independent experiments (**b**,**d**). * *p* < 0.05 vs. ctr, # *p* < 0.05 vs. TI. Significance was assessed by one-way ANOVA followed by Newman–Keuls test.

The ability of BAF-treated HMC3 cells to migrate towards the BBB led us to wonder whether microglia could modulate the BBB’s properties. We co-cultured an endothelial/astrocyte barrier with microglia cells. The endothelial layer was plated on 3 μm pore transwell inserts, astrocytes were plated on a coverslip, and microglia on the bottom of the well plate, in order to simulate the organization of the BBB and its interaction with microglia. Co-cultures were directly exposed to TI (10 UI and 5 UI) and to BAF (100 nM) + TI, and endothelial permeability to the 10 kDa FITC-conjugated dextran was evaluated after 24 h. Endothelial permeability to dextran was significantly increased by TI exposure, but BAF pretreatment reduced the cytokines’ effect (Figure 4a). Accordingly, BAF significantly prevented the TI-induced reduction in the expression of the tight junctional protein claudin-5 (Figure 4b).

To dissect the microglial effects on BBB properties, we pre-exposed microglia monocultures, plated on 24-well plates or 0.4 μm transwell inserts, to TI and BAF + TI (MG pre TI and MG pre BAF + TI, respectively). After 24 h, treatments were washed and pre-treated microglia were added to endothelial/astrocyte co-cultures (simulating the BBB). Triple co-cultures were then exposed to TI and BAF + TI. When cultures were exposed to TI in the presence of MG pre TI, endothelial permeability to the 10 kDA-conjugated dextran was increased (Figure 4c); conversely, when TI exposure occurred in the presence of microglia previously exposed to BAF and TI (MG pre BAF + TI), the cytokines’ effect on BBB permeability was significantly reduced compared to the condition in which microglia were previously exposed only to TI (MG pre TI) (Figure 4c). Exposure to BAF + TI in the presence of microglia pre-exposed to both TI and BAF + TI (MG pre TI and MG pre BAF + TI) reduced cytokine-induced barrier permeability, underlining the direct effect exerted by BAF in triple co-cultures (Figure 4c). Modifications in barrier permeability were mirrored by changes in the expression of claudin-5. Microglia’s preexposure to BAF + TI prevented the TI-induced reduction in claudin-5, and the expression of the protein was further maintained when the BBB was exposed to BAF + TI in the presence of either MG pre TI or MG pre BAF + TI (Figure 4d).

### 3.5. BAF-312 Modulates the Expression and Activity of MMP9 in Microglia

**Figure 5:**
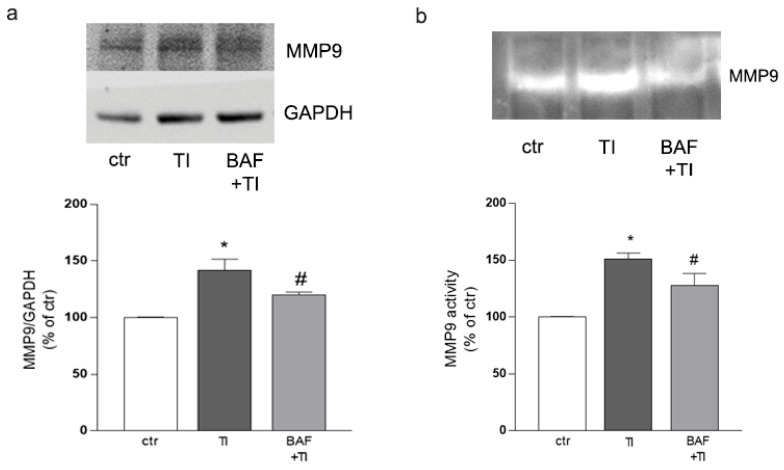
**MMP9 expression and activity is modulated by BAF-312.** HMC3, co-cultured with endothelial cells and astrocytes, were exposed to TI (10 UI, 5 UI) either alone or in the presence of BAF (100 nM). The expression of MMP9 (**a**) and its enzymatic activity (**b**) were evaluated after 24 h treatment. Representative blots and densitometric analysis are reported. Data are mean ± SEM of 3 independent experiments (**a**,**b**). * p < 0.05 versus ctr, # *p* < 0.05 vs. TI. Significance was assessed by one-way ANOVA followed by Newman–Keuls test.

Since claudin-5 is a substrate of the metalloprotease MMP9 [26], we evaluated the microglial expression and the enzymatic activity of this protease after exposure to TI (10 UI, 5 UI), either alone or in the presence of BAF (100 nM). TI significantly induced both the expression (Figure 5a) and the enzymatic activity (Figure 5b) of MMP9 as detected by Western blot and gel zymography, respectively, and BAF pretreatment significantly reduced these effects (Figure 5a,b).

## 4. Discussion

Data here reported highlight that, acting on microglia, BAF-312 is able to increase BBB stability. Microglia’s role in the CNS has been often associated with their ability to monitor the brain parenchyma, but also with the initiation and maintenance of inflammation. The role of microglia in supporting BBB stability has only recently emerged [14]. As a component of the NVU, microglia can directly communicate with the cerebral microvasculature, and resting microglia have been described in close proximity to cerebral microvessels in healthy rats [30]. Microglia, activated by a constant inflammatory stimulus, can contribute to BBB leakage, due to the release of inflammatory cytokines, and can also facilitate the invasion of circulating immune cells, by upregulating adhesion molecules [31,32,33]. However, at least in the initial phases of inflammation, perivascular microglia directly participate in BBB stability [12]. We have previously demonstrated that the stability of the BBB against an inflammatory stimulus can be enhanced by BAF-312, the recently approved modulator of S1P1 and S1P5 receptors [21,22]. Although the drug mainly prevents the egress of leukocytes from lymph nodes [34], it can easily cross the BBB and interacts with the different components of the CNS [24]. Microglia, as astrocytes, express S1P receptors [35], and here we demonstrated that BAF-312 can modulate specific microglia activities and their interaction with the cellular components of the BBB. In our study, microglial mobility appeared strongly related to the modulation of S1P1. Although BAF-312 acts on both S1P1 and S1P5, its selectivity for S1P1 is higher than for S1P5 (EC50 of 0.39 nM and 0.98 nM, for S1P1 and S1P5, respectively) [36]. Microglial migration was markedly reduced when S1P1 was blocked by the antagonist NIBR-0213 or in the presence of UC-42-WP04, which selectively activates S1P5, thus implying the different involvement of the two receptors in the modulation of microglial motility. BAF-312′s effects were indeed amplified in the presence of inflammatory cytokines. In our experimental protocol, while TI completely blocked HMC3 cell migration, this was rescued in the presence of BAF-312. Reduced microglial migration due to LPS and TI exposure has been previously described [37,38,39], a condition that could be reverted in the presence of the anti-inflammatory cytokine IL4 [39]. Accordingly, the anti–inflammatory effects exerted by BAF-312 on astrocytes [21,40] and on microglial cells [20,41] have been widely demonstrated.

Microglial mobility can be driven by matrix-degrading enzymes and, more specifically, the different involvement of MMP2 versus MMP9 has been described [37]. Thus, in our setting, we used either the broad-spectrum MMP inhibitor GM-6001 or the more specific MMP2 and MMP9 inhibitors, verifying that BAF-312′s effects on microglia movement strongly implicate MMP2; accordingly, both the gene expression and enzymatic activity of MMP2 in microglia exposed to inflammatory cytokines were strongly induced only in the presence of BAF-312. Microglia migration can be advantageous in the preservation of BBB functions in the early phases of inflammation [12], while impaired microglial motility—for example, that induced by the P2RY12 antagonist clopidogrel—can increase BBB injury [42]. We simulated in vitro microglial migration towards the BBB, to assess BAF-312′s effect in inflammatory conditions. We used two different models: (i) the scratch wound assay, in which microglia were only driven by the conditioned medium (CM) released by pretreated endothelial/astrocyte co-cultures, and (ii) the migration assay, where microglia were allowed to properly migrate towards the co-cultured BBB through transwell inserts. In both cases, microglia’s ability to migrate was reduced in the presence of inflammatory cytokines, while BAF-312 was able to restore migration, except when the CM was derived from an inflamed BBB. We then supposed that the BBB—and, more specifically, astrocytes—might release factors able to facilitate microglia’s movement towards the BBB. Again, this very likely involves MMP2, as the MMP2 inhibitor prevented BAF-312′s effects. The CCL5/CCR5 axis may drive microglial migration towards the perivascular space: in an in vivo model, aimed at evaluating microglial responses to systemic inflammation, blockade of CCL5 prevented microglial migration towards the BBB, enhancing barrier permeability during the early stages of inflammation [12]. Accordingly, in vitro, the pharmacological blockade of the CCL5 receptor CCR5, using maraviroc, was associated with reduced microglial migration only in the presence of glioma-soluble factors [43]. It has been also described that the CCL5/CCR5 axis increased cellular invasiveness via the induction of MMPs [44,45,46], and, more specifically, CCL5 modulated glioma cells’ migratory and invasive activities due to MMP2 upregulation [47]. We thus evaluated the gene expression of CCR5 on migrating microglia and of CCL5 on astrocytes. Results obtained suggest the involvement of the CCL5/CCR5 axis in our conditions, although activation of this pathway cannot be considered the only mechanism implied in microglia migration towards the BBB.

In our system, the increased migration of microglia in response to BAF appeared beneficial as it improved BBB functions. This is also supported by the effect observed on endothelial permeability that is strictly dependent on tight junctions’ stability. Accordingly, we observed that BAF counteracted the TI-induced increased permeability and restored claudin-5 expression. We previously demonstrated that endothelial/astrocyte exposure to BTI was able to reduce BBB damage, after 48 but not 24 h treatment [22]. Interestingly, we report here that, in the presence of microglia, the TI-reduced expression of claudin-5 was prevented already after 24 h of BAF exposure, probably due to the reduced expression and activity of microglial MMP9, of which claudin-5 is a substrate [26]. Our results confirm the active support given by microglia to BBB stability, as further strengthened by the observation that BAF-pretreated microglia are able to prevent TI-induced BBB damage.

## 5. Conclusions

In conclusion, these data support the hypothesis that BAF-312′s effects go beyond the mere regulation of leukocyte trafficking. In the CNS, BAF-312 can temper microglial activity and modulate its crosstalk with astrocytes, thus contributing to BBB stability.

## Data Availability

Not applicable.
